# Symplectic physics-embedded learning via Lie groups Hamiltonian formulation for serial manipulator dynamics prediction

**DOI:** 10.1038/s41598-025-17935-w

**Published:** 2025-09-26

**Authors:** Fei Wang, Liping Chen, Jianwan Ding

**Affiliations:** School of Mechanical Science and Engineering, Huazhong University of Science and Technology, Wuhan, 430074 China

**Keywords:** Lie group, Hamiltonian dynamics, Physics-embedded, Dynamics prediction, Serial manipulator, Engineering, Mechanical engineering

## Abstract

Accurate dynamic modeling is critical for advanced robotic control, yet conventional methods struggle with manipulator nonlinear complexity. While Hamiltonian neural networks leveraging Lie group symmetries improve physical consistency of the network, existing methods overlook key limitations: unconstrained sparsity in mass, dissipation, and control matrices; redundancy in mass network outputs; and lack of validation on multi-rigid-body systems. This paper proposes a symplectic physics-embedded learning approach (SPEL) based on Lie group Hamiltonian formulations for enhanced dynamics modeling of serial manipulators. By systematically encoding physical priors such as Lie group symmetries and Hamiltonian dynamics into neural network design, SPEL enforces sparsity in mass, dissipation, and control input matrices via physics-driven constraints and replaces input-independent matrix elements with trainable parameters. These mechanisms structurally optimize the network topology, significantly reducing output dimensionality while preserving physical consistency of the network. Experimental validation on simulated two-link and revolute-prismatic-revolute (RPR) manipulators, as well as a real 6-DOF manipulator, demonstrates that SPEL reduces over 52% of the parameters, enhances computational efficiency by more than 75%, and achieves higher prediction accuracy. Additionally, Symplectic Physics-Embedded Learning Kolmogorov-Arnold Networks (SPEL-KAN) reduce over 63% of the parameters and improve computational efficiency by more than 39%. This approach embeds geometric-mechanical principles into architectures, balancing efficiency with interpretable predictions.

## Introduction

Serial or articulated manipulator, renowned for their precision and adaptability, are increasingly employed in various industrial and domestic applications, particularly in small and medium-sized enterprises where tasks demand high accuracy and flexibility in dynamic and semi-structured environments^1–3^. These manipulators often utilize harmonic drives as joint reducers, introducing control challenges due to joint flexibility and significant nonlinearities in system dynamics^4^. In practical applications, dynamic parameters of manipulators are often affected by environmental factors (e.g., temperature variations, material aging, assembly errors, and external disturbances), leading to systematic deviations from theoretical design values and thus reducing the prediction accuracy of models based on nominal parameters. As a classic example of multi-rigid-body systems, serial manipulators consist of multiple rigid links connected by joints (revolute or prismatic), forming a kinematic chain that inherently involves complex interactions between bodies and constrains. Effective control and performance optimization of such robotic systems necessitate the development of accurate dynamic models capable of predicting and identifying these nonlinear behaviors^5^. Model prediction involves forecasting system outputs based on given inputs and data, while parameter identification aims to determine the system’s underlying structure and parameters using collected data^6^. The primary objective is to estimate model parameters through experimental data analysis, employing regression models and algorithms to minimize an objective function and accurately map inputs to outputs, reflecting actual system behavior^7^.

Traditional methods of dynamic learning, such as inverse dynamic models using ordinary least squares, assume a linear relationship between joint torques and inertial parameters, based on rigid links and negligible nonlinearities^8^. Although efficient for linear systems, these methods can yield biased and physically inconsistent results due to measurement noise, incorrect filtering, or inadequate excitation^9^.

Recent advances in machine learning address dynamic prediction challenges in robotic systems, where nonlinearities and uncertainties hinder traditional methods^10,11^. Machine learning models identify input-output relationships, often replacing physics-based approaches, but conventional neural networks lack physical interpretability and require substantial data. Physics-informed neural networks (PINNs) integrate physical constraints into neural network loss functions via automatic differentiation^12^, combining physics-driven and data-driven strengths to enhance nonlinear fitting accuracy and reduce data dependency^11^. Applications in fluid dynamics, solid mechanics, chemical kinetics, and mechanics demonstrate PINNs’ predictive capabilities comparable to conventional algorithms while enabling efficient parameter training and identification^13^.

However, PINNs struggle with complex systems governed by nonlinear, coupled ODEs with many parameters^14,15^, whereas physics-encoded neural networks (PeNNs) incorporate geometric constraints to reduce variance and accelerate convergence^16,17^. Physical system dynamics are governed by kinematic constraints and energy conservation, which can be enhanced via prior knowledge integration, including symmetry-equivariant networks^18^, graph networks for forward kinematics^19^, or Lagrangian/Hamiltonian architectures^20–24^. For serial manipulators adhering to Lagrangian/Hamiltonian mechanics, Greydanus et al. modeled Hamiltonians using neural networks to minimize symplectic gradient discrepancies^22^, with additional constraints that the mass matrix is positive definite and the dissipation matrix is positive semi-definite when employing Hamiltonian mechanics. Chen et al. and Zhong et al. applied adjoint methods for backpropagation through ODE solvers without explicit time derivatives^25,26^. Researchers like Yang^4^, Wu^27^, and Liu^28^ have developed Lagrangian/Hamiltonian-based manipulator models for system identification and control, with Yang neglecting joint coupling and Wu approximating uncertainties via standard deep neural networks to enhance Lagrangian neural networks.

Previous studies on Lagrangian/Hamiltonian neural networks often employed vector-valued states like Euler angles for manipulator modeling. In contrast, serial manipulators composed of rigid bodies inherently follow the Lie group structure, with kinematics evolving on the special Euclidean group SE(3) for spatial movements and the special orthogonal group SO(3) for rotations^29^. The exponential map from Lie group theory enables more efficient dynamic equations than traditional Denavit-Hartenberg (DH) methods. Recent advancements combine Hamiltonian mechanics with Riemannian geometry to derive concise inverse dynamic equations^30^, while Lie group Neural ODEs (NODEs) aim to preserve the Lie group structure during backpropagation in high-dimensional spaces or local coordinates^31^. Duong et al. proposed port-Hamiltonian NODEs (PHNODEs) on Lie groups for a quadrotor, establishing a novel framework for structure-preserving dynamic modeling^29,32^.

Recent advances in data-driven modeling include the introduction of Kolmogorov-Arnold Networks (KANs) by Liu et al. as an alternative to Multi-Layer Perceptrons (MLPs)^33,34^. KANs learn activation functions using gridded basis functions and trainable scaling factors, accelerating convergence, and reducing parameters. They have been applied in various fields, including time series prediction.

Even with Hamiltonian or Lagrangian frameworks, accurately acquiring all physical parameters of multi-rigid-body systems (e.g., serial manipulators) and precisely modeling complex nonlinear dissipative effects remain extremely challenging. In existing robotic dynamics modeling, while Featherstone’s ABA algorithm^35^ enables efficient computation via topological sparsity, and Murray^36^ revealed the underlying sparse structure of control matrices, both rely on analytical models and cannot learn nonlinear disturbances. Recent PINNs have attempted to introduce sparsity via L1 regularization but fail to incorporate robotics-specific geometric priors (e.g., Lie group structures). Existing Lagrangian or Hamiltonian neural networks for serial manipulator dynamics often rely on Euler angles, and neglect input-independent elements in the mass and control input matrices, leading to redundant outputs. While Hamiltonian neural networks on Lie groups offer improvements, they do not comprehensively model multi-rigid-body serial manipulators and fail to account for the inherent sparsity in mass, dissipation, and control input matrices. To address these limitations, this paper proposes a symplectic physics-embedded learning approach (SPEL) based on Lie group Hamiltonian formulations for dynamic prediction in serial manipulators. By systematically encoding physical priors, such as Lie group symmetries and Hamiltonian conservation laws, into the neural architecture, it enforces sparsity in system matrices via physical constraint-guided topology optimization and replaces input-independent matrix elements with trainable parameters to eliminate redundancy. These methods simplify network complexity while preserving physical consistency of the network, i.e., its ability to adhere to fundamental physical laws (e.g., energy conservation, momentum evolution, and structural sparsity of dynamic matrices) through the embedding of physical priors^37^.

The structure of the remainder of this paper is as follows: Section “Preliminaries” provides an overview of Hamiltonian dynamics on Lie groups and the PHNODEs approach. Section “Hamiltonian dynamics of serial manipulator on Lie groups” introduces the Hamiltonian dynamics of a serial manipulator on Lie groups. Section “Symplectic physics-embedded learning of Lie groups dynamics for serial manipulator” details the SPEL for a serial manipulator on Lie groups. Section “Experimental validation” presents the results from simulation and real-world experiments. Section “Conclusion” summarizes the paper and discusses future directions.

## Preliminaries

### Hamiltonian dynamics on Lie group

The pose of the body-fixed frame relative to the world frame is defined by the position $\varvec{r}_{\varvec{c}}\in \mathbb {R} ^{3}$ of the center of mass and the orientation of the body-fixed frame’s coordinate axes $\varvec{R} \in \textrm{SO}(3)$. The generalized coordinate $\varvec{q}$ is given by $\varvec{q}=(\varvec{r}_{\varvec{c}},\varvec{R})$. The rigid-body position and orientation can be combined into a single pose matrix $\varvec{T} \in \textrm{SE}(3)$. The kinematic equations of motion for a rigid body are defined by the linear velocity $\varvec{v} \in \mathbb {R} ^{3}$ and angular velocity $\varvec{\omega } \in \mathbb {R} ^{3}$ of the body-fixed frame relative to the world frame, both expressed in the body-frame coordinates. The generalized velocity $\varvec{\zeta } =(\varvec{v},\varvec{\omega }) \in \mathbb {R} ^{6}$ governs the rate of change of the rigid body’s pose, adhering to the principles of SE(3) kinematics^38^.

1 $$\begin{aligned} \dot{\varvec{q}} =\varvec{q\xi } =\varvec{q}\hat{\varvec{\zeta }} =\varvec{q}\begin{bmatrix} \hat{\varvec{\omega }} & \varvec{v}\\ 0^{T} & 0\end{bmatrix} \end{aligned}$$

where the symbol $\hat{}$ represents the mapping from a vector $\varvec{\zeta } \in \mathbb {R} ^{6}$ to a $4\times 4$ twist matrix $\varvec{\xi }=\hat{\varvec{\zeta }}$ in the Lie algebra $\mathfrak {se}(3)$ of SE(3), and from a vector $\varvec{\omega }$ to a $3\times 3$ skew-symmetric matrix $\hat{\varvec{\omega }}$ in the Lie algebra $\mathfrak {so}(3)$ of SO(3).

A port-Hamiltonian generalization of Hamiltonian mechanics is utilized to model systems that incorporate energy-storing elements (e.g., kinetic and potential energy), energy-dissipating elements (e.g., friction or resistance), and external energy sources (e.g., control inputs), interconnected through energy ports^29,39^.

2 $$\begin{aligned} \begin{bmatrix}\dot{\varvec{q}} \\ \dot{\varvec{p}}\end{bmatrix}=(\varvec{J}(\varvec{q},\varvec{p})-\varvec{R}(\varvec{q},\varvec{p}))\begin{bmatrix} \frac{\partial H}{\partial \varvec{q}} {}\\ \frac{\partial H}{\partial \varvec{p}} {} \end{bmatrix}+\varvec{G}(\varvec{q})\varvec{u} \end{aligned}$$

where $\varvec{J}(\varvec{q},\varvec{p})$ is a skew-symmetric interconnection matrix that represents the energy-storing elements, $\varvec{R}(\varvec{q},\varvec{p})\succeq 0$ is a positive semi-definite dissipation matrix that represents the energy-dissipating elements, and $\varvec{G}(\varvec{q})$ is an input matrix such that $\varvec{G}(\varvec{q})\varvec{u}$ represents the external energy sources. Energy-dissipating elements, such as friction or drag forces, are typically modeled as a positive semi-definite $\varvec{R}(\varvec{q},\varvec{p})\succeq 0$ and affect only the generalized momenta $\varvec{p}$, i.e.,

3 $$\begin{aligned} \varvec{R}(\varvec{q},\varvec{p})=\begin{bmatrix} 0 & 0\\ 0 & \varvec{D}(\varvec{q},\varvec{p})\end{bmatrix}, \varvec{J}(\varvec{q},\varvec{p})=\begin{bmatrix} 0 & \varvec{I}\\ -\varvec{I} & 0 \end{bmatrix}, \varvec{G}(\varvec{q})=\begin{bmatrix} 0 \\ \varvec{B}(\varvec{q}) \end{bmatrix} \end{aligned}$$

When the system is not subjected to external forces, meaning the system conserves energy, where the dissipation matrix is $\varvec{R}(\varvec{q}, \varvec{p}) = \varvec{0}, \varvec{u} = \varvec{0}$. By vectorizing the generalized coordinates $$\varvec{q}=\begin{bmatrix} \varvec{r}_{\varvec{c}}^{T}&\varvec{r}_{1}^{T}&\varvec{r}_{2}^{T}&\varvec{r}_{3}^{T}\end{bmatrix}^{T}$$, the variables of the port-Hamiltonian dynamics on SE(3) can be represented using the following interconnection matrix.

4 $$\begin{aligned} \varvec{q}^{\times } =\begin{bmatrix} \varvec{R}^{T} & 0 & 0 & 0\\ 0 & \hat{\varvec{r}}_{\varvec{1}}^{T} & \hat{\varvec{r}}_{\varvec{2}}^{T} & \hat{\varvec{r}}_{\varvec{3}}^{T} \end{bmatrix}, \varvec{p}^{\times } =\begin{bmatrix} 0 & \hat{\varvec{p}}_{\varvec{v}} \\ \hat{\varvec{p}}_{\varvec{v}} & \hat{\varvec{p}}_{\varvec{\omega } }\end{bmatrix} \end{aligned}$$

5 $$\begin{aligned} \varvec{J}(\varvec{q},\varvec{p})=\begin{bmatrix} 0 & \varvec{q}^{\times } \\ -\varvec{q}^{\times T} & \varvec{p}^{\times }\end{bmatrix}, \varvec{D}(\varvec{q},\varvec{p})=\begin{bmatrix} \varvec{d}_{\varvec{v}}(\varvec{q},\varvec{p}) & 0 \\ 0 & \varvec{d}_{\varvec{\omega }}(\varvec{q},\varvec{p})\end{bmatrix} \end{aligned}$$

where the elements $\varvec{d}_{\varvec{v}}(\varvec{q},\varvec{p})$ and $\varvec{d}_{\varvec{\omega }}(\varvec{q},\varvec{p})$ correspond to linear momentum $\varvec{p}_{\varvec{v}}$ and angular momentum $\varvec{p}_{\varvec{\omega }}$, respectively. The port-Hamiltonian’s equations on the SE(3) manifold are^29,39,40^:

6 $$\begin{aligned} \left\{ \begin{array}{ll}\begin{aligned}\dot{\varvec{r}_{\varvec{c}}}=& \varvec{R}\frac{\partial H(\varvec{q},\varvec{p})}{\partial \varvec{p}_{\varvec{v}}} \\ \dot{\varvec{r}_{i}}=& \varvec{r}_{i}\times \frac{\partial H(\varvec{q},\varvec{p})}{\partial \varvec{p}_{\varvec{\omega }}},i=1,2,3 \\ \dot{\varvec{p}_{\varvec{v}}}=& \varvec{p}_{\varvec{v}}\times \frac{\partial H(\varvec{q},\varvec{p})}{\partial \varvec{p}_{\varvec{\omega }}}-\varvec{R}^{T}\frac{\partial H(\varvec{q},\varvec{p})}{\partial \varvec{r}_{\varvec{c}}}-\varvec{d}_{\varvec{v}}(\varvec{q},\varvec{p})\frac{\partial H(\varvec{q},\varvec{p})}{\partial \varvec{p}_{\varvec{v}}}+\varvec{b}_{\varvec{v}}(\varvec{q})\varvec{u} \\ \dot{\varvec{p}_{\varvec{\omega }}}=& \varvec{p}_{\varvec{\omega }}\times \frac{\partial H(\varvec{q},\varvec{p})}{\partial \varvec{p}_{\varvec{\omega }}}+\varvec{p}_{\varvec{v}}\times \frac{\partial H(\varvec{q},\varvec{p})}{\partial \varvec{p}_{\varvec{v}}} +\sum _{i=1}^{3}\varvec{r}_{i}\times \frac{\partial H(\varvec{q},\varvec{p})}{\partial \varvec{r}_{i}}-\varvec{d}_{\varvec{\omega }}(\varvec{q},\varvec{p})\frac{\partial H(\varvec{q},\varvec{p})}{\partial \varvec{p}_{\varvec{\omega }}} +\varvec{b}_{\varvec{\omega }}(\varvec{q})\varvec{u} \end{aligned}\end{array}\right. \end{aligned}$$

where the input matrix is $$\varvec{B}(\varvec{q})=\begin{bmatrix} \varvec{b}_{\varvec{v}}(\varvec{q})^{T}&\varvec{b}_{\varvec{\omega }}(\varvec{q})^{T} \end{bmatrix}^{T}$$.

### Port-Hamiltonian neural ODE networks on Lie group

Port-Hamiltonian Neural ODE Networks on Lie Group integrates Neural ODE with Port-Hamiltonian systems^29^. It learns the Port-Hamiltonian function $H(\varvec{q},\varvec{p})$ on Lie group, providing dynamics through Hamilton’s equations. By embedding Hamiltonian dynamics on Lie group into the neural network model $\bar{f} (\varvec{x},\varvec{\theta } )$ and considering zero-order hold control inputs *u*, this network extends Neural ODEs, resulting in a system described by:

7 $$\begin{aligned} \begin{bmatrix} \dot{\varvec{x}} \\ \dot{\varvec{u}}\end{bmatrix}=\begin{bmatrix}f(\varvec{x},\varvec{\theta } )\\ 0 \end{bmatrix}=\bar{f} (\varvec{x},\varvec{\theta } ) \end{aligned}$$

The loss function for this network resembles mean squared error (MSE) but is computed between the predicted and actual generalized coordinates and velocities. The predicted coordinates $\bar{\varvec{q}}^{(i)}_{n}=(\bar{\varvec{r}}_{\varvec{c}n}^{(i)},\bar{\varvec{R}}_{n}^{(i)})$ and the ground-truth coordinates $\varvec{q}^{(i)}_{n}=(\varvec{r}_{\varvec{c}n}^{(i)},\varvec{R}_{n}^{(i)})$ are used to compute a loss on the Lie group manifold.

8 $$\begin{aligned} L(\varvec{\theta })=\sum _{i=1}^{D}\sum _{n=1}^{N}\left\| \log _{\textrm{SO}(3)}^{\vee }{\left( \bar{\varvec{R}}_{n}^{(i)}\varvec{R}_{n}^{(i)} \right) } \right\| _{2}^{2}+\sum _{i=1}^{D}\sum _{n=1}^{N}\left\| \varvec{r}_{\varvec{c}n}^{(i)}-\bar{\varvec{r}}_{\varvec{c}n}^{(i)} \right\| _{2}^{2}+\sum _{i=1}^{D}\sum _{n=1}^{N}\left\| \varvec{\zeta }_{n}^{(i)}-\bar{\varvec{\zeta }}_{n}^{(i)} \right\| _{2}^{2} \end{aligned}$$

where *D* represents the number of configurations $\varvec{q}$ in the dataset, and *N* denotes the length of the time series.

## Hamiltonian dynamics of serial manipulator on Lie groups

### Dissipative function and potential energy

Analyzing Eq. (2), in practical scenarios, control inputs are typically precise and predictable, whereas the $\varvec{D}(\varvec{q},\varvec{p})$ term exerts a nonlinear influence on the system’s behavior. This component accounts for various uncertainties in the serial manipulator, which mainly include the following factors.

Friction forces: Robot joint friction, including static and dynamic friction, introduces errors and can be modeled using Coulomb and viscous friction models. Elastic forces: Robot components, such as linkages and transmissions, can deform elastically under torque, generating opposing forces. Nonlinear dynamic effects: The manipulator may exhibit nonlinear behaviors like bending, complex periodic motions, and chaos. External disturbance forces: External forces, such as wind, vibrations, or impacts, can disrupt manipulator motion. Sensor errors: Sensor inaccuracies, including noise and drift, can lead to errors in the dynamics equations. Other model errors: Mathematical models may not fully capture real-world conditions, leading to errors from approximations and unmodeled dynamics^27^.

For the joint friction in the serial manipulator, the actual joint module comprises components such as a servo torque motor, joint position encoder, and joint reducer. The complex structure of these joints complicates the joint model, affecting the dynamic performance of the robot. Joint friction primarily includes bearing and brush friction in the motor, gear friction in the reducer, nonlinear elastic deformation between the flexible and rigid wheels of the harmonic drive reducer, and bearing friction at the joint. Several methods exist for calculating joint friction in serial manipulators.

9 $$\begin{aligned} {F_{s} = f_{s}\cdot e^{(-\zeta /\zeta _{s})}},F_{v} =-B\cdot \zeta \end{aligned}$$

where $F_{s}$ is Stribeck friction force; $f_{s}$ is Stribeck friction coefficient; $\zeta _{s}$ is characteristic velocity; $F_{v}$ is viscous friction; *B* is the coefficient of viscous friction. Therefore, $\varvec{D}(\varvec{q},\varvec{p})$ in this paper needs to be established as a neural network of $\varvec{q}$ and $\varvec{p}$, or a neural network of $\varvec{q}$ and $\varvec{\zeta }$.

The gravitational potential energy $V(\varvec{q})$ depends on the generalized coordinate $\varvec{q}$ of the serial manipulator and is influenced by the mass of the links and rotors. It can be used for its calculation, incorporating the gravity vector *g*, the total mass of each link and rotor, and their respective position vectors in the base coordinate system.

### Specifics of serial manipulator dynamics on Lie groups

A serial manipulator consists of a series of links connected by joints, which can be either revolute or prismatic. These joints are typically arranged sequentially along a single axis. In a serial manipulator, the motion of each joint influences the position and orientation of the end-effector. The manipulator’s movement is controlled by adjusting the angles or positions of the individual joints to execute specific tasks, such as handling, assembly, welding, and more.

In this section, the serial manipulator’s state is defined by the generalized coordinates $\varvec{q}=(\varvec{r}_{\varvec{c}},\varvec{R}), \varvec{r}_{\varvec{c}}\in \mathbb {R}^{3}, \varvec{R}\in \textrm{SO}(3)$ evolving on the Lie group G and the generalized velocity $\varvec{\zeta } =(\varvec{v},\varvec{\omega } )\in \mathbb {R}^{6}$ in the Lie algebra $\mathfrak {g}$ of G. The following diagram illustrates a schematic of a particular link in a serial manipulator.

**Figure 1:**
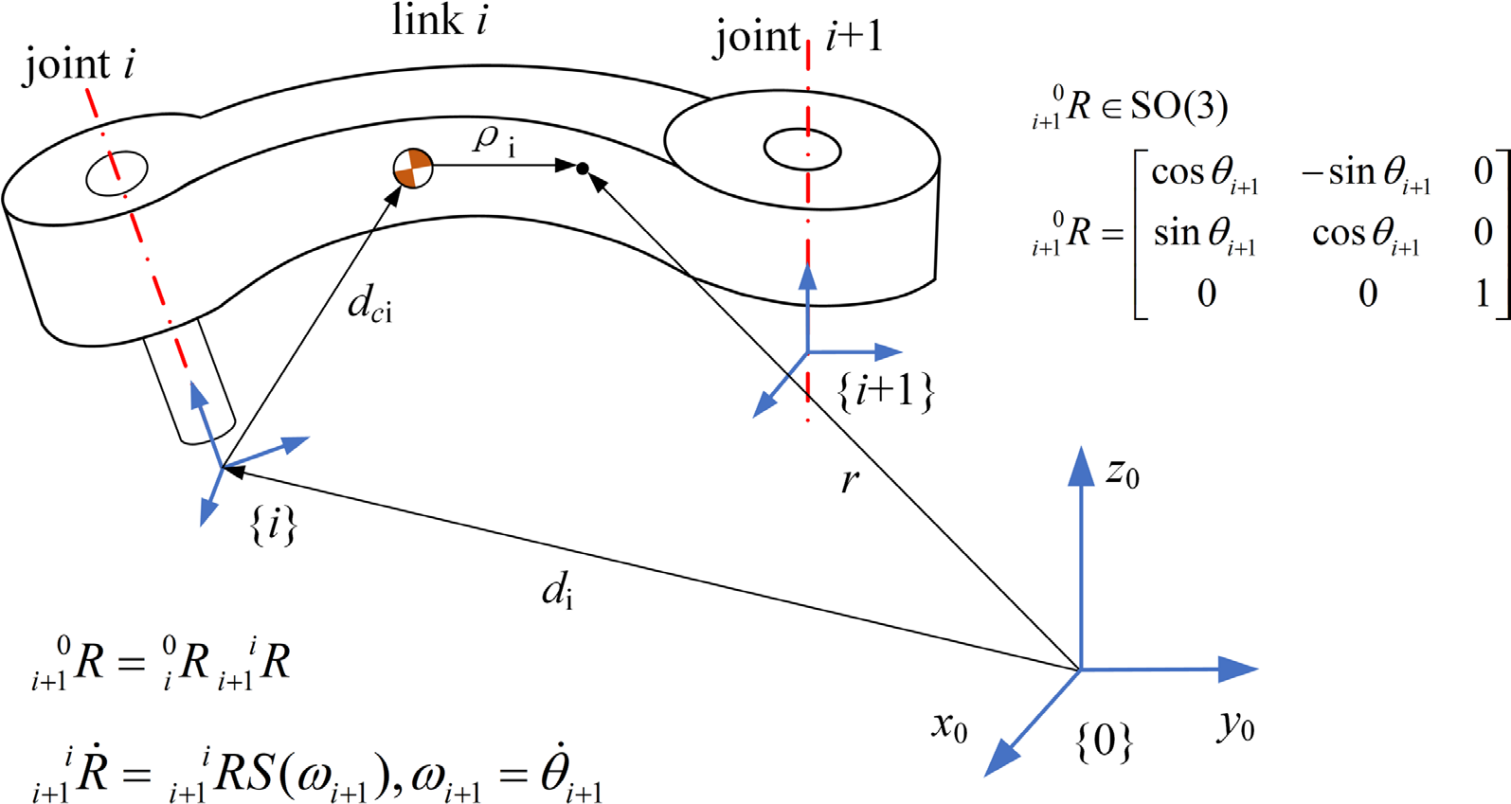
A particular link in a serial manipulator.

As shown in Fig. 1, {0} denotes the base coordinate system, and {*i*} represents the joint coordinate system attached to the *i*-th joint. Let $_{i}^{0}\varvec{R}\in \textrm{SO}(3)$ be the rotation matrix from the *i*-th joint coordinate system to the base coordinate system, and let $\varvec{d}_{i}\in \mathbb {R}^{3}$ be the position vector of the *i*-th joint in the base coordinate system, which can be obtained through the homogeneous transformation matrix. Let $\varvec{d}_{\varvec{c}i}\in \mathbb {R}^{3}$ be the vector from the *i*-th joint to the center of mass of the *i*-th link, mi be the scalar mass of the *i*-th link, and $\varvec{\rho }_{i}\in \mathbb {R}^{3}$ be the vector from the center of mass of the *i*-th link to the mass element, expressed in the *i*-th joint coordinate system. The vector from the origin of the base coordinate system to the mass element is given by $\varvec{d}_{i}+_{i}^{0} \varvec{R}(\varvec{d}_{\varvec{c}i}+\varvec{\rho }_{i})$.

For the *i*-th link with prismatic freedom, $\varvec{\omega }_{i}=0$. Thus, the kinetic energy and the potential energy of the *i*-th link are^39^

10 $$\begin{aligned} \begin{aligned} T_{i}(\varvec{R}_{i},\varvec{d}_{\varvec{c}i},\varvec{\omega }_{i})&=\frac{1}{2} \int _{B_{i}}\left\| \dot{\varvec{d}_{i}}+_{i-1}^{0}\dot{\varvec{R}}(\varvec{d}_{\varvec{c}i}+\varvec{\rho }_{i})+_{i-1}^{0}\varvec{R}(\dot{\varvec{d}}_{\varvec{c}i}+\varvec{\rho }_{i}) \right\| ^{2}dm(\varvec{\rho }_{i}) \\&=\frac{1}{2}m_{i}\left\| \dot{\varvec{d}}_{i} \right\| ^{2}+\frac{1}{2}\varvec{\omega }_{i}^{T}\varvec{J}_{i}\varvec{\omega }_{i}+\frac{1}{2}m_{i}\dot{\varvec{d}_{\varvec{c}i}}^{T}\dot{\varvec{d}}_{\varvec{c}i}+m_{i}\dot{\varvec{d}_{i}}^{T}\ _{i-1}^{0}\varvec{R}\left[ S(\varvec{\omega }_{i-1})\varvec{d}_{\varvec{c}i}+\dot{\varvec{d}}_{\varvec{c}i} \right] \\ &\quad +m_{i}\varvec{d}_{\varvec{c}i}^{T}S^{T}(\varvec{\omega }_{i-1})\dot{\varvec{d}}_{\varvec{c}i} \end{aligned} \end{aligned}$$

11 $$\begin{aligned} \begin{aligned} V_{i}(\varvec{R}_{i},\varvec{d}_{\varvec{c}i})=m_{i}g({\varvec{e}} _{3}^{T}\varvec{d}_{i}+{\varvec{e}}_{3}^{T}\ _{i}^{0}\varvec{R}\varvec{d}_{\varvec{c}i}) \end{aligned} \end{aligned}$$

where $\varvec{J}_{i}=m_{i}\varvec{S}^{T}(\varvec{d}_{\varvec{c}i})\varvec{S}(\varvec{d}_{\varvec{c}i})+\int _{B_{i}}\varvec{S}^T(\varvec{\rho }_{i})\varvec{S}(\varvec{\rho }_{i})dm(\varvec{\rho }_{i})$ is the $3\times 3$ inertia matrix of the *i*-th object; $\varvec{\omega }_{i-1}$ is the angular velocity of the $(i-1)$-th link relative to the base coordinate frame, and $_{i-1}^{0}\dot{\varvec{R}}=_{i-1}^{0}\varvec{RS}(\varvec{\omega }_{i-1})$; $\varvec{S}(\varvec{\omega }_{i-1})$ denotes the skew-symmetric matrix. The xy-plane of the base coordinate frame serves as the zero potential energy reference. The total kinetic energy *T* and total potential energy *V* of the serial manipulator are

12 $$\begin{aligned} \begin{aligned} T(\varvec{R},\varvec{d_{c}},\varvec{\omega },\dot{\varvec{d}}_{\varvec{c}})=\frac{1}{2} \sum _{i=1}^{n}\begin{bmatrix}[ m_{i}\left\| \dot{\varvec{d}}_{i} \right\| ^{2}+2m_{i}\dot{\varvec{d}}_{i}^{T}\ _{i-1}^{0}\varvec{R}\left[ \varvec{S}(\varvec{\omega }_{i-1})\varvec{d}_{\varvec{c}i}+\dot{\varvec{d}}_{\varvec{c}i} \right] \\ +\varvec{\omega }_{i}^{T}\varvec{J}_{i}\varvec{\omega }_{i}+m_{i}\dot{\varvec{d}}_{\varvec{c}i}^{T}\dot{\varvec{d}}_{\varvec{c}i}+2m_{i}\varvec{d}_{\varvec{c}i}^{T}\varvec{S}^{T}(\varvec{\omega }_{i-1})\dot{\varvec{d}}_{\varvec{c}i}\end{bmatrix} \end{aligned} \end{aligned}$$

13 $$\begin{aligned} \begin{aligned} V(\varvec{R},\varvec{d})=\sum _{i=1}^{n}m_{i}g({\varvec{e}}_{3}^{T}\varvec{d_{i}}+{\varvec{e}}_{3}^{T}\ _{i}^{0}\varvec{R}\varvec{d}_{\varvec{c}i}) \end{aligned} \end{aligned}$$

The Lagrangian $L(\varvec{q},\varvec{\zeta })$ of the manipulator is defined as $L(\varvec{q},\varvec{\zeta })=T-V$. By applying the Legendre transformation, the generalized momentum $\varvec{p}_{\dot{\varvec{d}}_{\varvec{c}i}}$ is defined as:

14 $$\begin{aligned} \begin{aligned} \varvec{p}_{\dot{\varvec{d}}_{\varvec{c}i}}=\frac{\partial L(\varvec{q},\varvec{\zeta })}{\partial \dot{\varvec{d}}_{\varvec{c}i}}, \varvec{p}=\frac{\partial L(\varvec{q},\varvec{\zeta })}{\partial \varvec{\zeta }}=M\varvec{\zeta } \end{aligned} \end{aligned}$$

where $\varvec{p}=\left[ \varvec{p}_{1}\dots \varvec{p}_{\dot{\varvec{d}}_{\varvec{c}i}}\dots \varvec{p}_{n} \right] ^{T},\varvec{\zeta }=\left[ \varvec{\zeta }_{1}\dots \dot{\varvec{d}}_{\varvec{c}i}\dots \varvec{\zeta }_{n} \right] ^{T},\varvec{q}=\left[ \varvec{q}_{1}\dots \varvec{d}_{\varvec{c}i}\dots \varvec{q}_{n} \right] ^{T}$; M denotes the mass matrix of the serial manipulator. Therefore, the Hamiltonian function is given by $H(\varvec{q},\varvec{p})=\varvec{p}^T \cdot \varvec{\zeta }-L(\varvec{q},\varvec{\zeta })=T+V$. For the *i*-th link with prismatic joints, the Hamiltonian canonical equations for the *i*-th joint are:

15 $$\begin{aligned} \begin{aligned} \left\{ \begin{array}{ll}\begin{aligned}\dot{\varvec{d}}_{\varvec{c}i}& =\frac{\partial H(\varvec{q},\varvec{p})}{\partial \varvec{p}_{\dot{\varvec{d}}_{\varvec{c}i}}} \\ \varvec{p}_{\dot{\varvec{d}}_{\varvec{c}i}} & = -\frac{\partial H(\varvec{q},\varvec{p})}{\partial \varvec{r}_{\varvec{c}}}-D_{\dot{\varvec{d}}_{\varvec{c}i}}(\varvec{q},\varvec{p})\frac{\partial H(\varvec{q},\varvec{p})}{\partial \varvec{p}_{\dot{\varvec{d}}_{\varvec{c}i}}}+\varvec{b}_{\dot{\varvec{d}}_{\varvec{c}i}}(\varvec{q})\varvec{u} \end{aligned}\end{array}\right. \end{aligned} \end{aligned}$$

For the *i*-th link with a revolute degree of freedom, the linear velocity $\varvec{v}=0$, $\varvec{d}_{\varvec{c}i}$ is a constant vector, and $_{i}^{i-1} \varvec{R}$ is not the identity matrix. The time derivative of the rotation matrix $_{i}^{i-1}\dot{\varvec{R}} =_{i}^{i-1}\varvec{RS}(\varvec{\omega }_{i})$, $\varvec{w}_{i}$ is the angular velocity of the *i*-th link in the *i*-th joint coordinate system. The position vector from the origin of the inertial frame to the mass element is given by $d_{i} +_{i}^{0}\varvec{R} (\varvec{d}_{\varvec{c}i} +\varvec{\rho } _{i})$. Thus, The potential energy of the *i*-th link is given by Eq. (11), and the kinetic energy is^39^:

16 $$\begin{aligned} \begin{aligned} T_{i}(\varvec{R}_{i},\varvec{d}_{i},\varvec{\omega }_{i})&=\frac{1}{2} \int _{B_{i}}\left\| \dot{\varvec{d}_{i}}+_{i-1}^{0}\dot{\varvec{R}}(\varvec{d}_{\varvec{c}i}+\varvec{\rho }_{i}) \right\| ^{2}dm(\varvec{\rho }_{i}) \\&=\frac{1}{2}m_{i}\left\| \dot{\varvec{d}}_{i} \right\| ^{2}+\frac{1}{2}\varvec{\omega }_{i}^{T}\varvec{J}_{i}\varvec{\omega }_{i}+m_{i}\dot{\varvec{d}}_{i}^{T}\ _{i}^{0}\varvec{RS}(\varvec{\omega }_{i})\dot{\varvec{d}}_{\varvec{c}i} \end{aligned} \end{aligned}$$

The total potential energy is given by Eq. (13), and the total kinetic energy is

17 $$\begin{aligned} \begin{aligned} T(\varvec{R},\varvec{d},\varvec{\omega })=\frac{1}{2} \sum _{i=1}^{n}\left[ m_{i}\left\| \dot{\varvec{d}}_{i} \right\| ^{2}+\varvec{\omega }_{i}^{T}\varvec{J}_{i}\varvec{\omega }_{i}+2m_{i}\dot{\varvec{d}}_{i}^{T}\ _{i}^{0}\varvec{RS}(\varvec{\omega }_{i})\varvec{d}_{\varvec{c}i} \right] \end{aligned} \end{aligned}$$

The generalized momentum $\varvec{p}_{\varvec{\omega }_{i}}$ is defined as $\varvec{p}_{\varvec{\omega }_{i}}=\partial L(\varvec{q},\varvec{\zeta })/{\partial \varvec{\omega }_{i}}$, $\varvec{p}=\left[ \varvec{p}_{1}\dots \varvec{p}_{\varvec{\omega }_{i}}\dots \varvec{p}_{n} \right] ^{T}$. The generalized velocity is $\varvec{\zeta }=\left[ \varvec{\zeta }_{1}\dots \varvec{\omega }_{i}\dots \varvec{\zeta }_{n} \right] ^{T}$. The generalized coordinates is $\varvec{q}=\left[ \varvec{q}_{1}\dots _{i}^{0}\varvec{R}\dots \varvec{q}_{n} \right] ^{T}$. For the *i*-th link with revolute joints, the Hamiltonian canonical equations for the *i*-th joint are:

18 $$\begin{aligned} \begin{aligned} \left\{ \begin{array}{ll}\begin{aligned}\dot{\varvec{r}}_{ij}& =\varvec{r}_{ij}\times \frac{\partial H(\varvec{q},\varvec{p})}{\partial \varvec{p}_{\varvec{\omega }_{i}}},j=1,2,3 \\ \dot{\varvec{p}}_{\varvec{\omega }_{i}} & = \varvec{p}_{\varvec{\omega }_{i}}\times \frac{\partial H(\varvec{q},\varvec{p})}{\partial \varvec{p}_{\varvec{\omega }_{i}}}+\sum _{j=1}^{3}\varvec{r}_{ij}\times \frac{\partial H(\varvec{q},\varvec{p})}{\partial \varvec{r}_{ij}}-\varvec{D}_{\varvec{\omega }_{i}}(\varvec{q},\varvec{p})\frac{\partial H(\varvec{q},\varvec{p})}{\partial \varvec{p}_{\varvec{\omega }_{i}}}+\varvec{b}_{\varvec{\omega }_{i}}(\varvec{q})\varvec{u} \end{aligned}\end{array}\right. \end{aligned} \end{aligned}$$

If a link has both prismatic and rotational degrees of freedom, then the Hamiltonian equations for that link are given by Eq. (6).

## Symplectic physics-embedded learning of Lie groups dynamics for serial manipulator

### Framework overview

**Figure 2:**
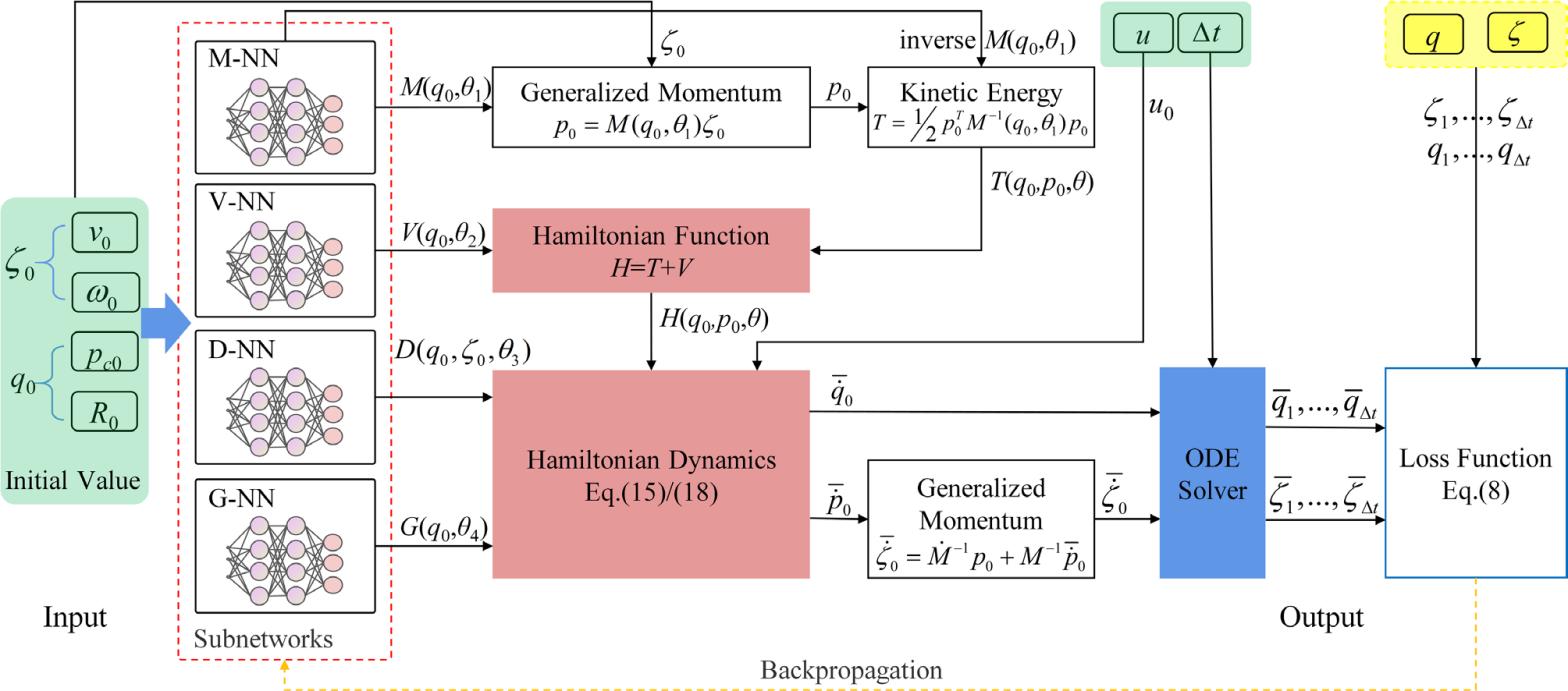
Architecture of SPEL on Lie group: the red part represents a critical component of the framework, wherein physical constraints are encoded into the neural network architecture; the green part denotes initial values; the yellow part indicates sequence values.

The dynamics of serial manipulators, governed by nonlinear and dynamically coupled ODEs with numerous parameters, represent a complex system. PeNNs adjust architectures by embedding specific physical constraints to ensure compliance during forward computation, thereby enhancing convergence speed and accuracy. This study introduces a SPEL framework based on Lie groups for predicting the dynamics of serial manipulators, as illustrated in Fig. 2.

In this method, neural networks model continuous ODEs using sequential data as input, employing ODE solvers to control approximation errors and facilitate flexible training, while learning the unknown parts of the Hamiltonian function and dissipation matrix that depend on the system states ($\varvec{q}$ and $\varvec{\zeta }$). The neural network comprises mass networks (M-NN), dissipation networks (D-NN), control input networks (G-NN), and potential energy networks (V-NN), collectively constructing the system’s dynamic state equations. By embedding the state-space dynamic model into the neural network and integrating the system’s mass and inertia into the state equations, accurate predictions can be achieved without acceleration data.

Algorithm 1 outlines the process of inputting initial generalized coordinates $\varvec{q}_{0}$ and velocities $\varvec{\zeta }_{0}$ into SPEL. Using subnetwork outputs for the mass matrix, dissipation matrix, potential energy, and control input matrix, the Hamiltonian function is derived from mass and potential energy. Generalized coordinates and momenta derivatives are computed via automatic differentiation with respect to $\varvec{q}$ and $\varvec{p}$, followed by solving the Hamiltonian equations. Given that generalized momenta $\varvec{p}$ are typically not directly measurable, their derivatives are converted into generalized accelerations $\dot{\varvec{\zeta }}$ using the relationship between $\varvec{p}$ and $\varvec{\zeta }$. An ODE solver, initialized with $\varvec{q}_{0}$ and $\varvec{\zeta }_{0}$, computes the predicted values of $\bar{\varvec{q}}_{1},...,\bar{\varvec{q}}_{\Delta t}$ and $\bar{\varvec{\zeta }}_{1},...,\bar{\varvec{\zeta }}_{\Delta t}$ at time $\Delta t$. These predictions are compared with actual values to calculate the loss, along with a penalty loss for the positive definiteness of the mass matrix, which is then used for backpropagation to update and optimize the neural network parameters.

**Figure:**
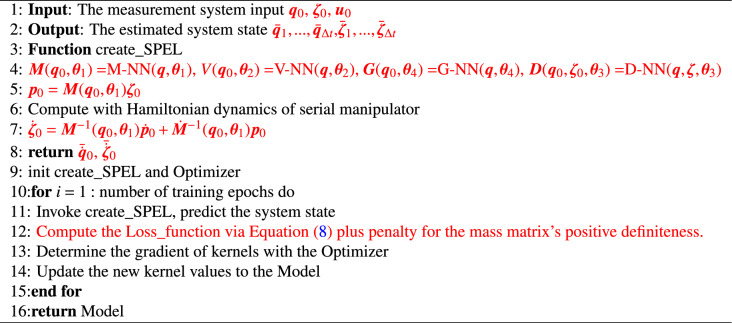
**Algorithm 1** SPEL on Lie group.

### Physics-embedded sparse subnetwork optimization

Physical constraints can be imposed on the parameters learned by the subnetworks. Specifically, the mass matrix must be positive definite, and the dissipation matrix must be positive semi-definite. These properties can be ensured using Cholesky decomposition, which represents each matrix as the product of a lower triangular matrix and its transpose, confirming their symmetry. Thus, only the diagonal elements and either the upper or lower triangular elements need to be specified to define the entire matrix.

**Figure 3:**
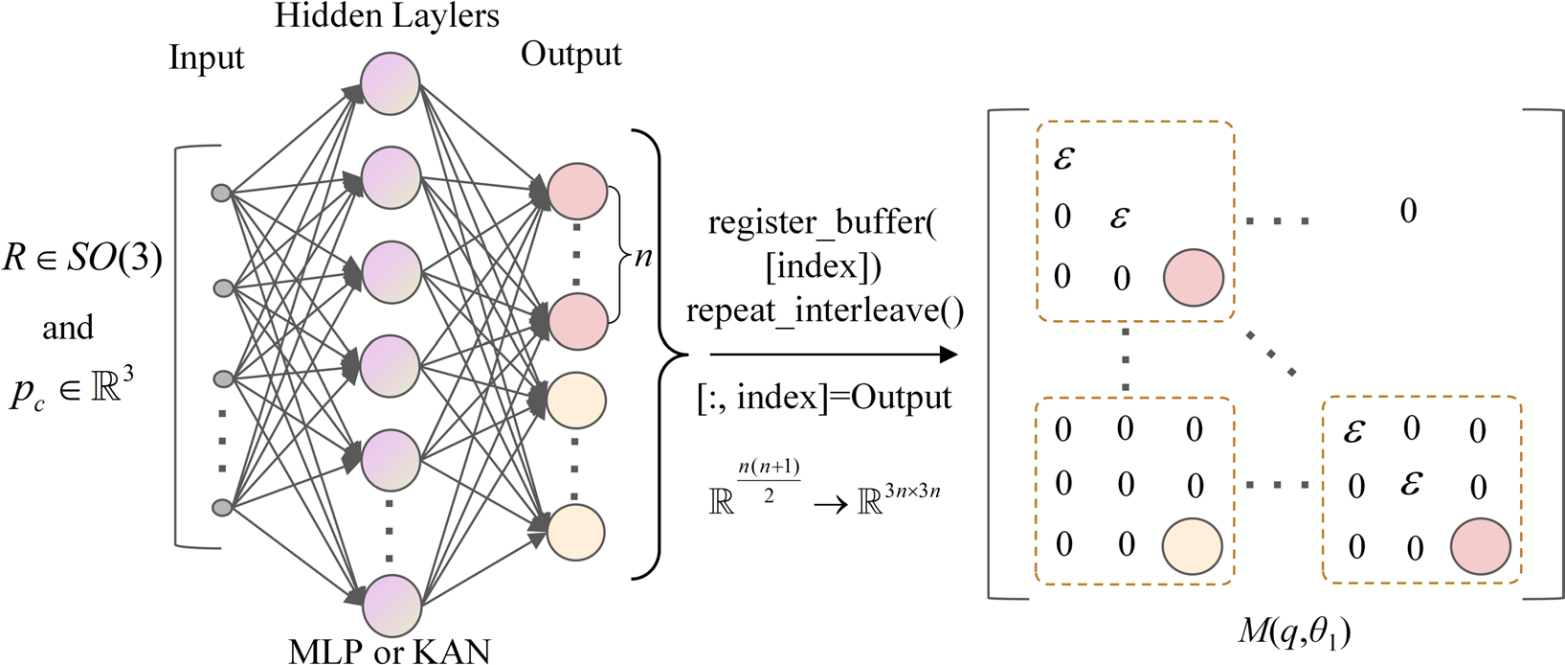
Simplification of M-NN: $\varepsilon$ is a small positive constant; *n* is the number of diagonal elements in the lower triangular matrix.

In the context of serial manipulator dynamics, when considering rotation about or translation along the z-axis, the generalized velocities are expressed as angular velocity $$\varvec{\omega } =\begin{bmatrix} 0&0&\omega _{1}\end{bmatrix}^{T}$$ and linear velocity $$\varvec{v} =\begin{bmatrix} 0&0&v_{1}\end{bmatrix}^{T}$$. Therefore, in practical computations, only the elements in the last row and column of the $3\times 3$ mass matrix on the Lie group are significant, while the other elements can be set to zero. For example, in Duong’s work^29^, the mass matrix in the Hamiltonian dynamics of an inverted pendulum on the Lie group is a $3\times 3$ matrix with only one unknown parameter, while the other elements are zero. This study embeds these physical insights into the mass network. To ensure the positive definiteness of the mass matrix, a small positive constant $\varepsilon$ is added to the diagonal elements, as shown in the Fig. 3. Therefore, the outputs of the mass network and the dissipative network are computed as $(n^{2}+n)/2$, where *n* is the number of diagonal elements in the lower triangular matrix, and the mass matrix is a $3n\times 3n$ matrix.

Similar considerations apply to the dissipation matrix in serial manipulator dynamics. Only the elements in the last row and column of the $3\times 3$ dissipation matrix on the Lie group are meaningful during calculations, while the remaining elements can be zero. Since the dissipation matrix is positive semi-definite, it does not require the addition of a small positive constant $\varepsilon$ to the diagonal elements.

**Figure 4:**
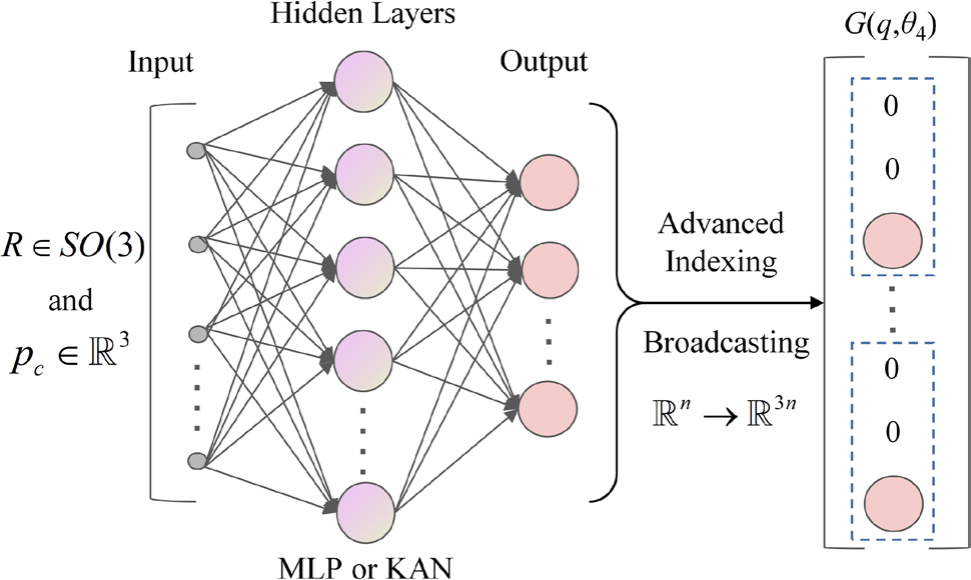
Simplification of G-NN: *n* is the number of output nodes.

The control input matrix is affected by the dynamic coupling between the joints, where each joint’s movement influences the others. Effective control requires considering the system’s overall state and making the control input matrix related to the coordinates of all joints. In practice, the control input matrix is often simplified by computing it only along the z-axis, similar to the mass matrix, as shown in Fig. 4.

The potential energy is computed using a simple, fully connected neural network with a single output.

### Network parameter conversion via physics-embedded outputs

For the constant values in the mass matrix and control input matrix, these constants are trained as parameters of the network rather than as output nodes^12^. Define the matrix form of the generalized mass matrix $\varvec{M}(\varvec{q})$ as

19 $$\begin{aligned} \varvec{M}(\varvec{q})=\begin{bmatrix} a+f_{1}(\varvec{q}) & ... & b+f_{2}(\varvec{q})\\ ... & & ... \\ b+f_{2}(\varvec{q}) & ... & c \end{bmatrix} \end{aligned}$$

where *a*, *b*, *c* are constants, and $f_{1}(\varvec{q})$, $f_{2}(\varvec{q})$ are functions of the generalized coordinates $\varvec{q}$, with all quantities *a*, *b*, *c*, $f_{1}(\varvec{q})$ and $f_{2}(\varvec{q})$ being scalar-valued. The *i*-th diagonal term of an n-axis manipulator’s mass matrix is the equivalent inertia of links *i* to *n* (all links 1 to *n*) relative to joint *i*. Revolute joints use the parallel axis theorem for inertia; prismatic joints use link masses. Non-diagonal (coupling) terms combine constants and joint position functions. The *n*-th diagonal term, position-independent, is *c*; the first, with position-dependent components, is $a + f_{1}(\varvec{q})$.

In the matrix $\varvec{M}(\varvec{q})$, only $f_{1}(\varvec{q})$ and $f_{2}(\varvec{q})$ depend on the input $\varvec{q}$. Constants *a*, *b*, and *c* are treated as network parameters, initialized with initial values, and optimized through forward computation and backpropagation. The functions $f_{1}(\varvec{q})$ and $f_{2}(\varvec{q})$ serve as the output nodes. This approach reduces the number of network parameters, simplifies the model, avoids redundant learning, improves training efficiency, enhances interpretability, and increases generalization. Specifically, for the mass matrix in the dynamics of the serial manipulator, this method can be applied to optimize the network structure and improve performance.

For example, the mass matrix $\varvec{M}_{rr}(\varvec{q})$ of a two-link (revolute-revolute) manipulator, as shown in Eq. (20), includes constants $\varvec{J}_{1}$ and $\varvec{J}_{2}$, which represent the moments of inertia and are defined as $\varvec{J}_{1}=m_{1}\varvec{S}^{T}({\varvec{e}}_{1}){\varvec{S}}({\varvec{e}}_{1})$ and $\varvec{J}_{2}=m_{2}\varvec{S}^{T}({\varvec{e}}_{1}){\varvec{S}}({\varvec{e}}_{1})$, where $m_{1}$ and $m_{2}$ are the masses of links. These constants are independent of the generalized coordinates of the manipulator. Similarly, the mass matrix $\varvec{M}_{rp}(\varvec{q})$ of a revolute-prismatic (RP) manipulator, as shown in Eq. (20), includes the moments of inertia $\varvec{J}_{1}$ and mass $m_{2}$, both of which are independent of the generalized coordinates. Therefore, these parameters can be iteratively optimized as part of the network’s parameters rather than being treated as output nodes.

20 $$\begin{aligned} \varvec{M}_{rr}(\varvec{q})=\begin{bmatrix} \varvec{J}_{1}+5\varvec{J}_{2}+4m(\varvec{q}) & \varvec{J}_{2}+2m(\varvec{q})\\ \varvec{J}_{2}+2m(\varvec{q}) & \varvec{J}_{2} \end{bmatrix},\varvec{M}_{rp}(\varvec{q})=\begin{bmatrix} \varvec{J}_{1} & 0\\ 0 & m_{2} \cdot ({\varvec{e}}_{3}{\varvec{e}}_{3}^{T}) \end{bmatrix} \end{aligned}$$

where $$m(\varvec{q})=m_{2}\varvec{S}^{T}({\varvec{e}}_{1})_{2}^{0}{\varvec{R}}^{T}\ _{1}^{0} {\varvec{RS}}({\varvec{e}}_{1})$$.

Equation (20) demonstrates that only part of the mass matrix for the two-link manipulator depends on the generalized coordinates. As a result, the mass network for the RP manipulator comprises zero output nodes and two network parameters, whereas the mass network for the two-link manipulator includes one output node and necessitates two additional network parameters.

## Experimental validation

The simulation experiments were conducted in Python 3.9.15 using the Robotics Toolbox^41^. For real-world experiments, a 6-DOF serial manipulator from Zhongke Shengu was used, controlled by a system developed with cSPACE. Software modules were developed using ARM Cortex-A and MATLAB/Simulink. The controller’s internal processor is the TI Sitara AM4376. Joint modules use incremental encoders with 20,000 lines for motor angle detection and absolute encoders with 17-bit resolution for joint angle measurement, achieving an angular resolution of $0.0045^{\circ }$. Algorithm training was performed on a system equipped with a GeForce RTX 4090 GPU and 24 GB of memory.

### Data acquisition

**Table 1:** DH parameters.

Manipulator	$\theta _{j}$($^\circ$)	$d_{j}$(m)	$a_{j}$(m)	$\alpha _{j}$($^\circ$)	$q^{-}$($^\circ$)	$q^{+}$($^\circ$)
Two-Link	$q_{1}$	0	1	0.0	$-180.0$	180.0
	$q_{2}$	0	1	0.0	$-180.0$	180.0
RPR	$q_{1}$	0.154	0	90.0	$-170.0$	170.0
	$-90$	$q_{2}$	0.0203	0.0	0.305	1.27
	$q_{3}$	0	0	0.0	$-170.0$	170.0
Real	$q_{1}$	122.3	0	0.0	$-179.0$	179.0
	$q_{2}$	0	0	90.0	$-152.0$	152.0
	$q_{3}$	0	-270	0.0	$-146.0$	146.0
	$q_{4}$	123.3	-253	0.0	$-179.0$	179.0
	$q_{5}$	107.1	0	90.0	$-179.0$	179.0
	$q_{6}$	99.1	0	$-90.0$	$-179.0$	179.0

Numerical simulations provide effectively infinite sampling frequency and zero noise, enabling the generation of multiple datasets to assess algorithm sensitivity across various parameter values. This study uses the Robotics Toolbox to model a two-link and a revolute-prismatic-revolute (RPR) manipulator, with their DH parameters and other parameters (mass, friction coefficients) detailed in Tables 1 and 2, respectively. In this work, following the experimental design in Zhong’s work^26^, the control input *u* for each link is set to one of $-2,-1,0,1,2$ at a sampling frequency of 20 Hz. Dynamic simulations start from 64 different initial positions, collecting position, velocity, and force data for each joint over 1 second, ensuring data remain within normal ranges. Angular coordinates are converted into rotation matrices using DH parameters, and translational coordinates are extended to three dimensions. The dataset for the two-link manipulator contains 11520 samples, while the dataset for the RPR manipulator includes 7680 samples. For training, the first 0.25 seconds of sequence data are used, with 50% of the dataset allocated to the training set and 50% to the test set. The control input matrix $\varvec{B}(\varvec{q})$ for the two-link manipulator is defined as $$\varvec{B}(\varvec{q})=[\begin{matrix} 0&0&1&0&0&1\end{matrix}]^{T}$$. For the RPR manipulator, the control input matrix $\varvec{B}(\varvec{q})$ is given by $$\varvec{B}(\varvec{q})=[\begin{matrix} 0&0&1&0&0&1&0&0&1\end{matrix}]^{T}$$.

**Table 2:** Model parameters.

Manipulator	Links	*m*(kg)	$\varvec{I}$(kg m$^{2}$)	$\varvec{r}$(m)	$J_{m}$ (kg m$^{2}$)	*B*(N s/m)	$\varvec{T_{c}}$(N)	*G*
Two-Link	Link1	1	[1,1,1,1,1,1]	[− 0.5,0,0]	0	$1.48\times 10^{-3}$	[0.395,− 0.435]	1
	link2	1	[1,1,1,1,1,1]	[− 0.5,0,0]	0	$8.17\times 10^{-4}$	[0.126,− 0.071]	1
RPR	Link1	5.01	[0.108,0.018,0.1,0,0,0]	[0,− 1.05,0]	2.19	$1.48\times 10^{-3}$	[0.395,− 0.435]	1
	Link2	4.25	[2.51,2.51,0.006,0,0,0]	[0,0,− 6.45]	0.782	$1.67\times 10^{-3}$	[0.126,− 0.071]	1
	Link3	1.08	[0.002,0.001,0.001,0,0,0]	[0,0.092,− 0.054]	0.106	$1.817\times 10^{-3}$	[0.126,− 0.171]	1

In addition to simulation data, this study uses experimental data from a real 6-DOF manipulator to validate the method’s effectiveness in dynamic prediction. PID control is applied to each joint with a sampling frequency of 500Hz and a control frequency of 125Hz; for PID control, the trajectory is a sinusoidal curve with a frequency of 1/8 Hz and an amplitude of $30^{\circ }$, with tracking errors ranging from $-0.4^{\circ }$ to $+0.4^{\circ }$. Six sets of different PID parameters are configured to ensure normal operation, collecting position and torque data for each joint. Collected position data are processed using a zero-phase filter to eliminate phase delay and reduce high-frequency noise, preserving signal phase information and improving signal quality. Velocity is calculated using a differential method. Angular coordinates are converted into rotation matrices using DH parameters. Data not conforming to the 4:1 ratio between sampling and control frequencies are excluded. For each set of PID parameters, the first 1,000 samples of position, velocity, and torque are selected to form a dataset of 24,000 samples. Seventy percent of this dataset is used for training and 30% for testing.

In the experiments, the following models are employed to obtain the dynamics for both the simulated and real manipulator on Lie groups: PHNODEs, symplectic physics-embedded learning-KAN (SPEL-KAN), and SPEL. The PHNODEs employ Cholesky decomposition for the mass network and the dissipation network without any additional modifications. The SPEL-KAN substitutes the MLP with KAN. All experiments adopt the AdamW optimizer with a learning rate of 0.001. The goal is to evaluate the accuracy and physical plausibility of the proposed methods. Performance is assessed using MSE based on training and testing trajectory errors, and the results are compared with other models to determine relative accuracy and effectiveness.

### Simulation results

#### Two-link manipulator

**Figure 5:**
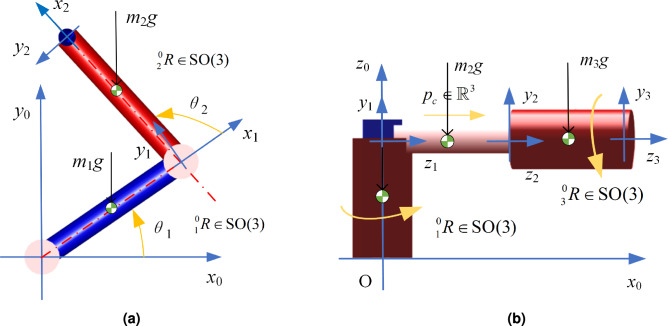
Schematic diagram of manipulator structure(3D visualization generated using MATLAB R2023a (

The two-link manipulator is shown in Fig. 5a. The manipulator operates in the xy-plane, with uniformly distributed material and mass along the links.

As shown in Fig. 5a, the equation of motion for the two-link manipulator on the Lie groups is given by:

21 $$\begin{aligned} _{1}^{0} \dot{\varvec{R}}=_{1}^{0} \varvec{RS}(\varvec{\omega } _{1}),_{2}^{0} \dot{\varvec{R}}=_{2}^{0} \varvec{RS}(\varvec{\omega } _{1}+\varvec{\omega } _{2}),_{2}^{0} \varvec{R}=_{1}^{0} \varvec{R}_{2}^{1} \varvec{R} \end{aligned}$$

22 $$\begin{aligned} _{1}^{0} \varvec{R}=\begin{bmatrix} \cos \theta _{1} & -\sin \theta _{1} & 0\\ \sin \theta _{1} & \cos \theta _{1} & 0\\ 0 & 0 & 1 \end{bmatrix},_{2}^{1} \varvec{R}=\begin{bmatrix} \cos \theta _{2} & -\sin \theta _{2} & 0\\ \sin \theta _{2} & \cos \theta _{2} & 0\\ 0 & 0 & 1 \end{bmatrix} \end{aligned}$$

where $\varvec{\omega }_{1}$ and $\varvec{\omega }_{2}$ represent the angular velocities of the links, given by $$\begin{bmatrix} 0&0&\dot{\theta _{1}} \end{bmatrix}^{T}$$ and $$\begin{bmatrix} 0&0&\dot{\theta _{2}} \end{bmatrix}^{T}$$, respectively, with the direction around the z-axis. $\varvec{S}(\varvec{\omega }_{1})$ and $\varvec{S}(\varvec{\omega }_{2})$ denote the skew-symmetric matrices corresponding to $\varvec{\omega }_{1}$ and $\varvec{\omega }_{2}$.

In the base coordinate frame, the center of mass of the first link is located at $\frac{1}{2}a_{1}\ _{1}^{0}\varvec{R}{\varvec{e}}_{1}$ and the center of mass of the second link is located at $a_{1}\ _{1}^{0}\varvec{R\textrm{e}}_{1}+\frac{1}{2} a _{2}\ _{2}^{0} {\varvec{R}\textrm{e}}_{1}$. $\textrm{e}_{1}$ is the unit vector $$\begin{bmatrix} 1&0&0 \end{bmatrix}^{T}$$. The Lagrangian function of the two-link manipulator is given by:

23 $$\begin{aligned} \begin{aligned} L(\varvec{R},\varvec{\omega }_{})=\frac{1}{2} m_{1}\left\| _{1}^{0}\dot{\varvec{R}}{\varvec{e}}_{1} \right\| ^{2}+ \frac{1}{2} m_{2}\left\| 2_{1}^{0}\dot{\varvec{R}}{\varvec{e}}_{1}+_{2}^{0}\dot{\varvec{R}}{\varvec{e}}_{1} \right\| ^{2} -m_{1}g{\varvec{e}}^{T}_{2}\ _{1}^{0}\varvec{R\textrm{e}}_{1}-m_{2}g{\varvec{e}}^{T}_{2}\left( 2 _{1}^{0}\varvec{R\textrm{e}}_{1}+_{2}^{0}\varvec{R\textrm{e}}_{1}\right) \end{aligned} \end{aligned}$$

The dynamic equations based on the Lagrangian formulation can be derived as follows:

24 $$\begin{aligned} \begin{aligned} \begin{bmatrix}\tau _{1} \\ \tau _{2}\end{bmatrix}=&\begin{bmatrix} \varvec{J}_{1}+5\varvec{J}_{2}+4m_{2}\varvec{S}^{T}({\varvec{e}}_{1})_{0}^{2}\varvec{R}^{T}\ _{1}^{0}\varvec{RS}({\varvec{e}}_{1}) & \varvec{J}_{2}+2m_{2}\varvec{S}^{T}({\varvec{e}}_{1})_{0}^{2}\varvec{R}^{T}\ _{1}^{0}\varvec{RS}({\varvec{e}}_{1})\\ \varvec{J}_{2}+2m_{2}\varvec{S}^{T}({\varvec{e}}_{1})_{0}^{2}\varvec{R}^{T}\ _{1}^{0}\varvec{RS}({\varvec{e}}_{1}) & \varvec{J}_{2}\end{bmatrix}\begin{bmatrix}\dot{\varvec{\omega }_{1}} \\ \dot{\varvec{\omega }_{2}}\end{bmatrix}\\&+\begin{bmatrix}-2m_{2}\ _{2}^{0}\varvec{R}^{T}\ _{1}^{0}\varvec{RS}({\varvec{e}}_{1})\varvec{\omega }_{2}\times \varvec{S}({\varvec{e}}_{1})\varvec{\omega }_{2}-4m_{2}\ _{2}^{0}\varvec{R}^{T}\ _{1}^{0}\varvec{RS}({\varvec{e}}_{1})\varvec{\omega }_{1}\times \varvec{S}({\varvec{e}}_{2})\varvec{\omega }_{2}\\ 2m_{2}\ _{2}^{0}\varvec{R}^{T}\ _{1}^{0}\varvec{RS}({\varvec{e}}_{1})\varvec{\omega }_{1}\times \varvec{S}({\varvec{e}}_{1})\varvec{\omega }_{1}\end{bmatrix}\\&+\begin{bmatrix}m_{2}g\ _{2}^{0}\varvec{R}^{T}{\varvec{e}}_{2}\times {\varvec{e}}_{1}-(m_{1}+2m_{2})g\ _{1}^{0}\varvec{R}^{T}{\varvec{e}}_{2}\times {\varvec{e}}_{1}\\ -2m_{2}g\ _{2}^{0}\varvec{R}^{T}{\varvec{e}}_{2}\times {\varvec{e}}_{1}\end{bmatrix} \end{aligned} \end{aligned}$$

where $$\varvec{J}_{1}=m_{1}\varvec{S}^{T}({\varvec{e}}_{1})\varvec{S}({\varvec{e}}_{1}),\varvec{J}_{2}=m_{2}\varvec{S}^{T}({\varvec{e}}_{1})\varvec{S}({\varvec{e}}_{1})$$.

The mass matrix is derived as shown in Eq. (20). The potential energy is as follows

25 $$\begin{aligned} V(\varvec{q})=-\frac{3}{2}m_{1}ga_{1} {\varvec{e}}_{2}^{T}{_{1}^{0}} {\varvec{R} \text {e}}_{1}-\frac{1}{2} m_{\text {2}}ga _{2}{\varvec{e}}_{2}^{T}{_{2}^{0}} {\varvec{R} \text {e}}_{1} \end{aligned}$$

In summary, in SPEL and SPEL-KAN, the mass network is designed with 1 output node and 18 input nodes, with $\varvec{J}_{1}$ and $\varvec{J}_{2}$ set as network parameters. The control input network is configured such that its values are also set as network parameters, with zero output nodes.

**Figure 6:**
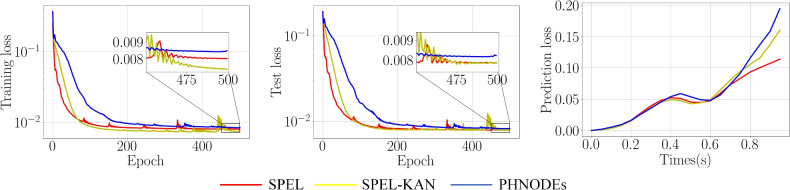
Losses for various network architectures on the two-link manipulator.

Based on Fig. 6 and Table 3, the performance of SPEL in learning the dynamics of a two-link manipulator can be evaluated. The subnetworks incorporate prior information about the manipulator’s dynamics, facilitating the learning of the underlying physical model. The proposed methods achieve equivalent modeling with fewer output nodes and network parameters, validating the effectiveness of the added physical constraints. The y Table S1 presents the obtained mass matrices and verifies their properties.

**Figure 7:**
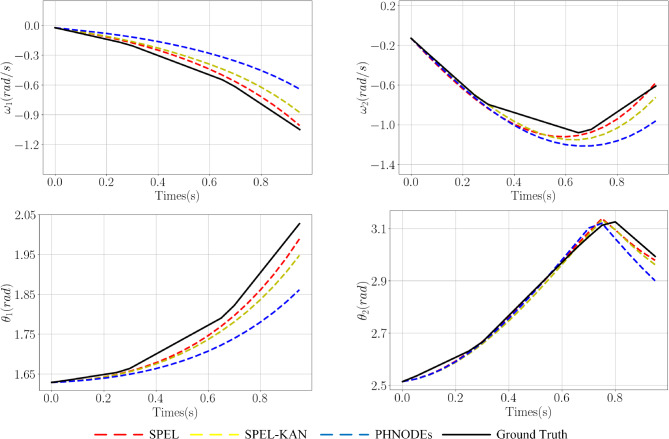
Comparison of predicted values for $\varvec{\omega }$ and $\theta$ across different networks in a two-link manipulator.

The model is trained using the first 0.25s of data and test set control inputs to predict state loss (for $\varvec{R}$ and $\varvec{\omega }$) over a 1s interval, thereby obtaining prediction loss. As illustrated in Table 3, Figs. 6, and 7, the proposed method reduces the number of output nodes, thereby lowering the dimensionality of dynamic parameter estimation and compressing the network parameter space, which in turn reduces model complexity. Specifically, SPEL and SPEL-KAN reduce the parameter counts to 20% and 5% of the original PHNODEs model, respectively. By minimizing the output nodes of M-NN and D-NN and eliminating G-NN, the method effectively removes redundant computations from the original model.

**Table 3:** Comparison of different neural network architectures for two-link manipulator dynamics learning.

	PHNODEs	**SPEL**	**SPEL-KAN**
M-NN Outputs	21	**1**	**1**
V-NN Outputs	1	1	1
D-NN Outputs	21	**3**	**3**
G-NN Outputs	6	**0**	**0**
Parameters	277,319	56,165	**13,604**
Activation function	tanh	tanh	learned
Training loss	$8.51\times 10^{-3}$	$8.01\times 10^{-3}$	$\varvec{7.39}\times \varvec{10^{-3}}$
Training time(s)	1.81	**0.31**	0.82
Prediction loss	0.194	**0.113**	0.160

1 -KAN indicates that the internal MLP has been replaced with a KAN.

2 M-NN Outputs, V-NN Outputs, D-NN Outputs, and G-NN Outputs denote the output nodes of the M-NN, V-NN, D-NN, and G-NN, respectively.

3 Training time includes the sum of forward computation time and backpropagation time

SPEL-KAN enhances efficiency by substituting fixed tanh activation functions with learnable ones, maintaining lower training errors even with only 24% of SPEL’s parameter count. This demonstrates significant parameter efficiency, potentially alleviating the need for manual network architecture design through adaptive feature extraction. Compared to fixed tanh activations, learnable activations reduce training loss by 7.7%, underscoring the importance of dynamically adjusting nonlinear features. However, this improvement introduces additional computational overhead, increasing training time from 0.31s to 0.82s. Despite achieving optimal training loss, SPEL-KAN exhibits higher prediction errors than SPEL, indicating potential overfitting risks. These findings offer new insights into lightweight network design, suggesting that dynamic activations can compensate for reduced network depth.

SPEL achieves the highest computational efficiency, running 5.8 times faster than PHNODEs, primarily because of its reduced parameter count and simplified computational graphs. In practical engineering applications with constrained hardware resources, SPEL optimally balances speed and accuracy. For scenarios demanding extremely lightweight models, developing specialized acceleration algorithms for SPEL-KAN is essential.

#### Revolute-prismatic-revolute manipulator

In this section, experiments are conducted using an RPR manipulator, as illustrated in Fig. 5b. The RPR manipulator, which includes an additional prismatic joint compared to the two-link manipulator, has the linear velocity of the second link denoted as *v*, directed along the z-axis. In the base coordinate frame, the center of mass of the first link is located at $\frac{1}{2}d_{1}\ _{1}^{0} \varvec{R\textrm{e}}_{3}$, the center of mass of the second link is located at $d_{1}\ _{1}^{0} \varvec{R\textrm{e}}_{3}+\frac{1}{2} q _{2}\ _{2}^{0} {\varvec{R}\textrm{e}}_{3}$, and the center of mass of the third link is located at $d_{1}\ _{1}^{0} \varvec{R\textrm{e}}_{3}+ q _{2}\ _{2}^{0} {\varvec{R}\textrm{e}}_{3}+\frac{1}{2} a _{3}\ _{3}^{0} {\varvec{R}\textrm{e}}_{3}$. The potential energy is as follows

26 $$\begin{aligned} V(\varvec{q}) & =-\frac{1}{2} m_{1}g d _{1}{\varvec{e}}_{3}^{T}\ _{1}^{0} {\varvec{R}\textrm{e}}_{3}- m _{2} g {{\varvec{e}}}_{3}^{T}( d _{1}\ _{1}^{0} {\varvec{R}\textrm{e}}_{3}+\frac{1}{2} q _{2}\ _{2}^{0} {\varvec{R}\textrm{e}}_{3}) \nonumber \\ & \quad - m _{3} g {\varvec{e}}_{3}^{T}( d _{1}\ _{1}^{0} {\varvec{R}\textrm{e}}_{3}+ q _{2}\ _{2}^{0} {\varvec{R}\textrm{e}}_{3}+\frac{1}{2} a _{3}\ _{3}^{0} {\varvec{R}\textrm{e}}_{3}) \end{aligned}$$

In the mass matrix of the RPR manipulator, based on the physical interpretation of the mass matrix, the last diagonal element corresponds to the moment of mass of the third link, as the third joint is a revolute joint. The middle diagonal element corresponds to the sum of the masses of the second and third links since the second joint is a prismatic joint.

In summary, in SPEL and SPEL-KAN, this work designs the mass network with 3 output nodes and 21 input nodes, with two elements on the diagonal set as network parameters. The control input network is designed with 3 output nodes and 21 input nodes. Consequently, the training results are shown in the following figure.

**Figure 8:**
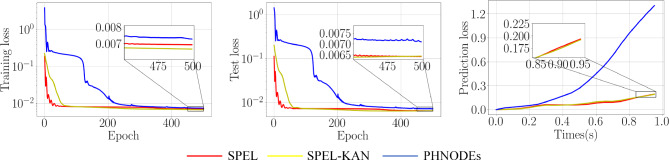
Losses for various network architectures on the RPR manipulator.

**Figure 9:**
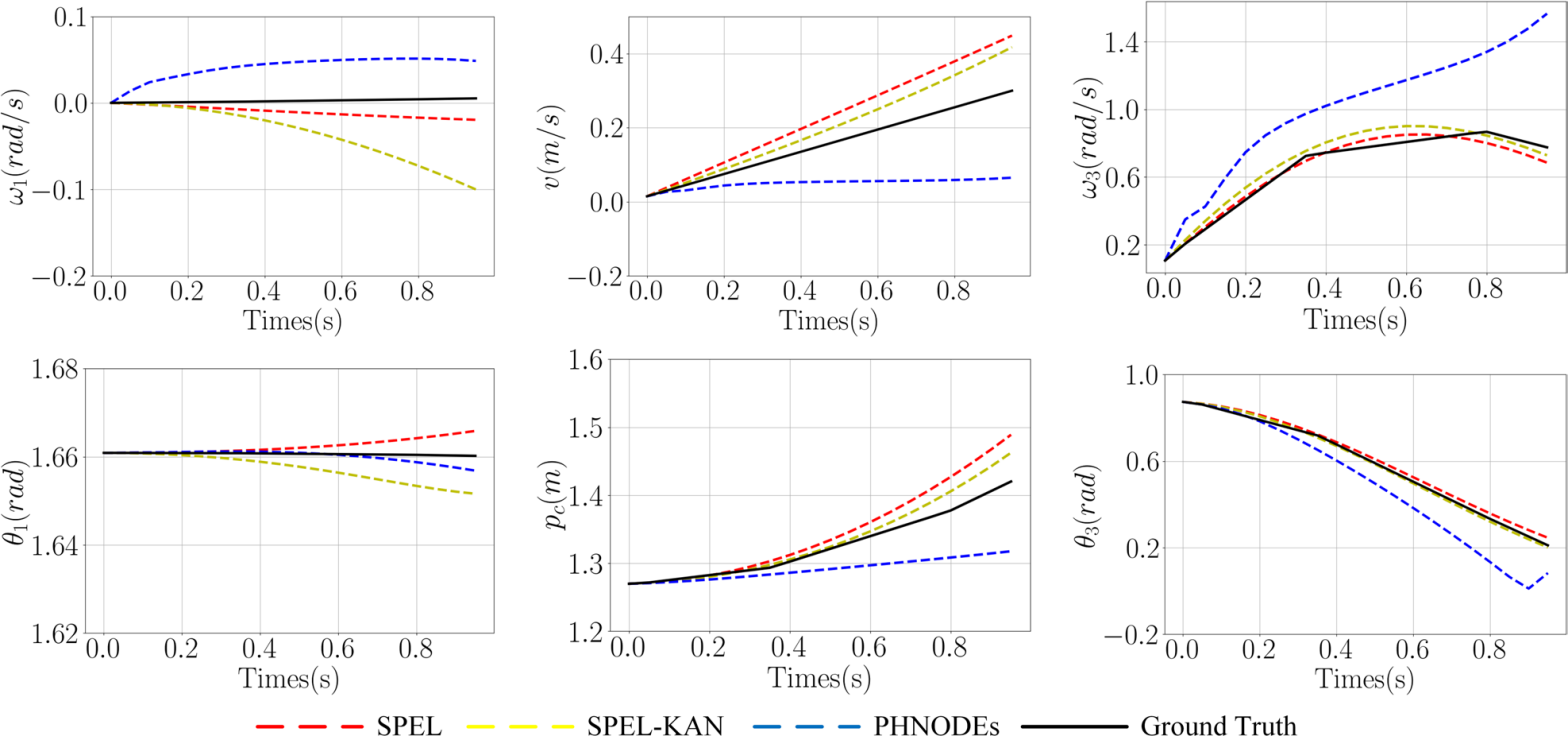
Comparison of predicted values for $\varvec{\omega }$ and $\theta$ across different networks in an RPR manipulator.

**Table 4:** Comparison of different neural network architectures for RPR manipulator dynamics learning.

	PHNODEs	SPEL	SPEL-KAN
M-NN Outputs	45	**3**	**3**
V-NN Outputs	1	1	1
D-NN Outputs	45	**6**	**6**
G-NN Outputs	9	**3**	**3**
Parameters	329,700	121,053	**118,812**
Activation function	tanh	tanh	learned
Training loss	$7.27\times 10^{-3}$	$6.92\times 10^{-3}$	$\varvec{6.65}\times \varvec{10^{-3}}$
Training time(s)	3.41	**0.71**	2.08
Prediction loss	1.30	0.193	**0.192**

1 -KAN indicates that the internal MLP has been replaced with a KAN.

2 M-NN Outputs, V-NN Outputs, D-NN Outputs, and G-NN Outputs denote the output nodes of the M-NN, V-NN, D-NN, and G-NN, respectively.

3 Training time includes the sum of forward computation time and backpropagation time

The model is trained using the first 0.25s of data and test set control inputs to predict state loss (for $\varvec{R}$ and $\varvec{\omega }$) over a 1s interval, thereby obtaining prediction loss. Based on Fig. 8 and Table 4, SPEL effectively learns the dynamics of the RPR manipulator, which includes both prismatic and revolute joints. With a smaller network structure and fewer parameters, SPEL incorporates prior information about the RPR manipulator’s dynamics into its subnetworks, enabling the learning of the underlying physical model and demonstrating superior modeling performance.

The proposed methods achieve equivalent modeling with fewer output nodes and network parameters, validating the effectiveness of the added physical constraints. Specifically, the process reduces the number of output nodes compared to the original PHNODEs model by integrating physical priors deeply into the network. This approach focuses on modeling manipulator dynamics rather than full-state prediction through physical constraints such as sparse matrices and constant elements, thereby compressing the network parameter space. Consequently, model complexity and trainable parameters are reduced, with SPEL and SPEL-KAN having 36.7% and 36% of the parameters of the original model, respectively.

In terms of computational efficiency, SPEL exhibits superior performance with the shortest training time, achieving a speedup of 4.8 times compared to PHNODEs. This improvement results from reduced backpropagation computation and simplified computational graphs. Although the training time for SPEL-KAN increases to 2.08 seconds due to the additional computational overhead from optimizing learnable activation functions, its parameter efficiency remains competitive. These methods are particularly significant for real-time online learning scenarios, with SPEL being suitable for high-frequency updates and SPEL-KAN more appropriate for offline optimization scenarios.

As illustrated in Figs. 8 and 9, prediction loss decreases from 1.30 in PHNODEs to 0.193 for SPEL and 0.192 for SPEL-KAN, marking an accuracy improvement of approximately 6.7 times. This reduction in prediction loss is significantly greater than the improvement in training loss (8.5% vs. 85%), indicating that the models more effectively capture the essential dynamics.

The use of learnable activations in KAN enhances nonlinear expression capabilities and improves dynamic feature extraction, resulting in better generalization. Although this optimization introduces increased computational complexity, it allows for a more compact parameter distribution. By trading off some training speed, KAN achieves higher modeling accuracy and shows a slight advantage during the prediction phase.

### Real 6-DOF manipulator

**Figure 10:**
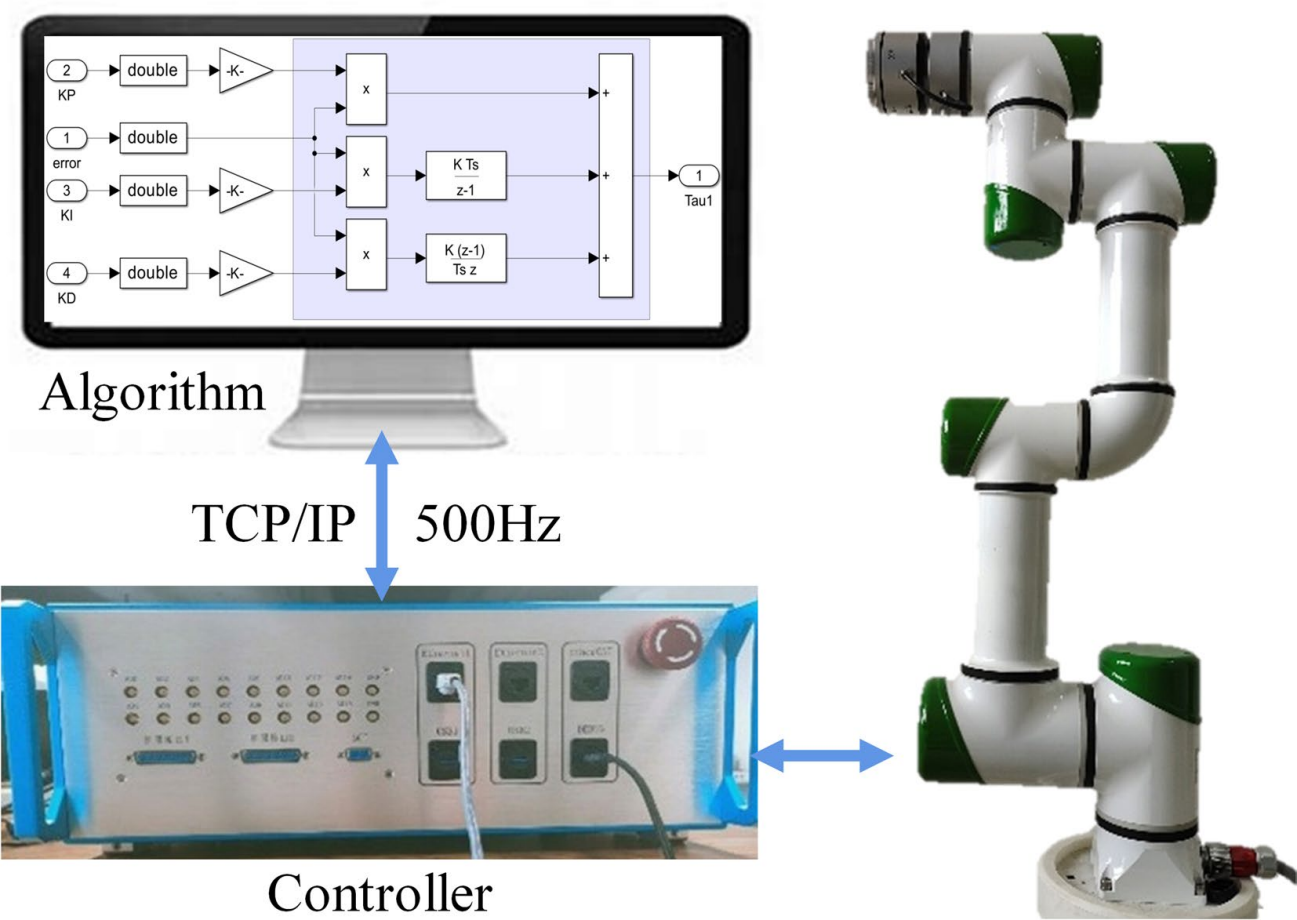
Real 6-DOF manipulator(Graphical reconstruction by authors based on original macrophotography ([iqoo neo5], [2025.02.03]), with dimensional enhancement via Microsoft PowerPoint 2021(

In this section, experiments are conducted using a real 6-DOF manipulator. The real 6-DOF manipulator has six rotational degrees of freedom, as shown in the Fig. 10. The DH parameters for this manipulator are provided in Table 1.

**Figure 11:**
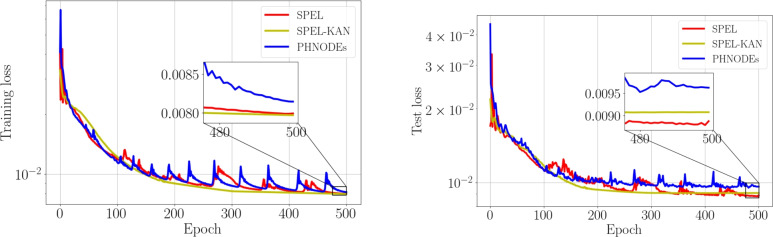
Losses for various network architectures applied to the real manipulator.

In SPEL and SPEL-KAN, the mass network has 20 output nodes, whereas, in PHNODEs, it has 171 output nodes. For the control input network, both SPEL and SPEL-KAN are designed with 6 output nodes, compared to 18 output nodes in PHNODEs. Similarly, the dissipation network in SPEL and SPEL-KAN has 21 output nodes, while PHNODEs have 171 output nodes. Consequently, the training losses are as shown in the Fig. 11. In the training loss curves, PHNODE exhibits the most severe oscillations, followed by SPEL, while SPEL-KAN shows a nearly flat curve. PHNODE’s unconstrained network outputs for mass, dissipation, and control matrices render it sensitive to noise from joint friction and sensor errors. SPEL reduces overfitting by compressing the parameter space with physical constraints, and SPEL-KAN further smooths fluctuations via learnable activation functions. Despite PHNODE converging, SPEL and SPEL-KAN demonstrate more stable convergence, highlighting the role of physical constraints in enhancing optimization stability and generalization, validating the study’s approach.

As shown in Table 5, the proposed method reduces redundant computations and matches the independent matrix elements by embedding physical priors and applying constraints like sparse matrices and constant elements. This compresses the network parameter space, reducing model complexity. SPEL and SPEL-KAN achieve respective parameter reductions of 52.1% and 73.8% compared to PHNODEs, thereby improving parameter efficiency and reducing memory usage.

SPEL achieves a 4.7-fold reduction in training time due to simplified gradient flows within physics-constrained architectures. In contrast, SPEL-KAN’s training time is 1.7 times longer than SPEL, resulting from the computational overhead associated with learning activation functions, yielding a minimal improvement in training loss of 0.38%. The time-to-accuracy ratio of SPEL-KAN stands at 17.0 seconds per 0.01 loss reduction, compared to 3.8 seconds for SPEL, posing questions regarding its suitability for online learning scenarios.

While SPEL achieves the lowest test loss, SPEL-KAN’s higher test error despite lower training loss reveals a 14.0% overfitting gap, up from SPEL’s 9.8%. This implies that KAN’s flexible activations may overfit noise in dynamics data, though its parameter efficiency partially mitigates this through inherent regularization.

**Table 5:** Comparison of different neural network architectures for real manipulator dynamics learning.

	PHNODEs	SPEL	SPEL-KAN
M-NN Outputs	171	**20**	**20**
V-NN Outputs	1	1	1
D-NN Outputs	171	**21**	**21**
G-NN Outputs	18	**6**	**6**
Parameters	326,911	156,359	**85,521**
Activation function	tanh	tanh	learned
Training loss	$8.13\times 10^{-3}$	$7.99\times 10^{-3}$	$\varvec{7.96}\times \varvec{10^{-3}}$
Training time(s)	17.70	**3.76**	6.44
Test loss	$9.62\times 10^{-3}$	$\varvec{8.77}\times \varvec{10^{-3}}$	$9.07\times 10^{-3}$

1 -KAN indicates that the internal MLP has been replaced with a KAN.

2 M-NN Outputs, V-NN Outputs, D-NN Outputs, and G-NN Outputs denote the output nodes of the M-NN, V-NN, D-NN, and G-NN, respectively.

3 Training time includes the sum of forward computation time and backpropagation time

**Figure 12:**
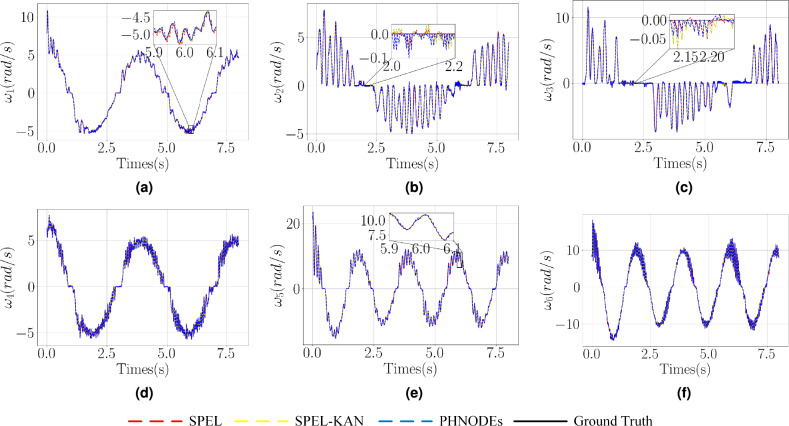
Prediction results of various networks for $\varvec{\zeta }$ with experimental data.

**Figure 13:**
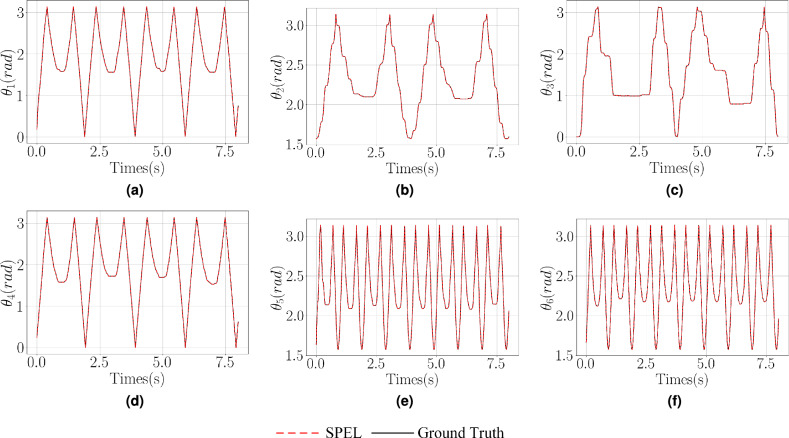
Prediction results of SPEL for $\varvec{q}$ with experimental data.

**Table 6:** The data in the table represents the MSE between the actual and predicted values of $\varvec{\zeta }$ and $\varvec{q}$ for each joint.

	Model	Joint 1	Joint 2	Joint 3	Joint 4	Joint 5	Joint 6	Sum
$\varvec{\zeta }$	PHNODEs	$1.90\times 10^{-3}$	$4.90\times 10^{-3}$	$8.60\times 10^{-3}$	$6.30\times 10^{-3}$	$1.08\times 10^{-2}$	$4.69\times 10^{-2}$	$7.93\times 10^{-2}$
	**SPEL**	$\varvec{1.10}\times \varvec{10^{-3}}$	$2.10\times 10^{-3}$	$\varvec{3.20}\times \varvec{10^{-3}}$	$\varvec{3.40}\times \varvec{10^{-3}}$	$\varvec{4.30}\times \varvec{10^{-3}}$	$1.04\times 10^{-2}$	$\varvec{2.45}\times \varvec{10^{-2}}$
	**SPEL-KAN**	$1.30\times 10^{-3}$	$\varvec{2.00}\times \varvec{10^{-3}}$	$3.40\times 10^{-3}$	$3.80\times 10^{-3}$	$4.40\times 10^{-3}$	$\varvec{1.01}\times \varvec{10^{-2}}$	$2.50\times 10^{-2}$
$\varvec{q}$	PHNODEs	$6.67\times 10^{-9}$	$2.00\times 10^{-4}$	$8.08\times 10^{-8}$	$5.55\times 10^{-8}$	$2.30\times 10^{-3}$	$2.20\times 10^{-3}$	$4.707\times 10^{-3}$
	**SPEL**	$4.13\times 10^{-9}$	$2.00\times 10^{-4}$	$\varvec{1.45}\times \varvec{10^{-8}}$	$\varvec{1.51}\times \varvec{10^{-8}}$	$2.30\times 10^{-3}$	$2.20\times 10^{-3}$	$4.693\times 10^{-3}$
	**SPEL-KAN**	$\varvec{4.05}\times \varvec{10^{-9}}$	$2.00\times 10^{-4}$	$3.61\times 10^{-8}$	$2.00\times 10^{-8}$	$2.30\times 10^{-3}$	$2.20\times 10^{-3}$	$\varvec{4.691}\times \varvec{10^{-3}}$

1 -KAN indicates that the internal MLP has been replaced with a KAN

Figure 12 presents the predicted joint velocities for joints 1-6 of the serial manipulator across various networks, while Fig. 13 shows the prediction joint positions of joints 1-6 using the SPEL network. As shown in Table 6, quantifying the prediction errors for the dynamics of a 6-DOF manipulator, SPEL reduces overall errors by 69.1% in $\varvec{\zeta }$ predictions compared to PHNODEs, with the greatest improvement (77.8% reduction) observed in joint 6 under high dynamic loads. This validates the precise modeling capability of physical constraints for complex end-effector dynamics. Although SPEL-KAN slightly increases total error by 2.0%, it maintains the lowest error in joint 6, indicating that the KAN structure enhances nonlinear feature extraction through learnable activation functions.

In $\varvec{q}$ predictions, SPEL-KAN achieves the best performance, representing a 0.34% reduction compared to PHNODEs. Notably, the error for low-inertia joint 1 decreases by 39.3%, highlighting the synergistic effect of physical constraints and adaptive activations—where physical constraints reduce overfitting risk by compressing the parameter space, while adaptive activations improve the capture of high-frequency signals.

## Conclusion

This study proposes a SPEL method for serial manipulators on Lie groups, achieving precise dynamic prediction by integrating physical information directly into a nonlinear ordinary differential equation system. By analyzing Hamiltonian dynamics on Lie groups, the research presents a port-Hamiltonian dynamics model for the multi-rigid-body serial manipulator that incorporates Lie group kinematics and dynamic constraints.

The analysis emphasizes the sparse matrix properties of the mass, dissipation, and control input matrices. Incorporating these structures into neural network design minimizes output layer nodes and trainable parameters through leveraging known constant information. Enhancements to the dissipation network address joint friction, while the introduction of KAN further improves accuracy and reduces trainable parameters via learnable activation functions, extending KAN’s applicability to complex systems.

Evaluations using data from a two-link manipulator and an RPR manipulator, along with data from a real 6-dof manipulator, confirm high predictive accuracy and computational efficiency. Compared to PHNODEs, both SPEL and SPEL-KAN reduce output nodes and trainable parameters, simplify the computational graph, and improve convergence rates. SPEL shows faster computational efficiency, while SPEL-KAN has lower training losses and fewer trainable parameters. These results highlight SPEL’s advantages in parameter reduction and convergence rate, making it a preferred choice for robotic applications.

Despite these achievements, the model’s performance depends heavily on the accuracy of integrated physical information. Although significant reductions in trainable parameters and improvements in convergence speed were observed, further optimization of input layer nodes is required for maximum efficiency. Future work will focus on minimizing the number of input layer nodes in SPEL on Lie groups, conducting thorough comparisons with traditional methods, and exploring applications in parameter identification. These efforts aim to enhance model efficiency and deepen understanding of underlying dynamics, thereby advancing robotic control and dynamic modeling.

## Supplementary Information

Supplementary Information.

## Data Availability

The data supporting the findings of this study are available from the corresponding author upon request.
