# Supplementary material for: Symplectic physics-embedded learning via Lie groups Hamiltonian formulation for serial manipulator dynamics prediction
Source: Sci Rep. 2025 Sep 26;15:33179. doi: 10.1038/s41598-025-17935-w (PMC12475180; doi:10.1038/s41598-025-17935-w)
Supplement: Supplementary file 1 — Supplementary Information. [file 41598_2025_17935_MOESM1_ESM.pdf]

Supplementary material

Symplectic Physics-Embedded Learning via Lie Group Hamiltonian Formulation for Serial Manipulator Dynamics Prediction

Fei Wang[1], Liping Chen[1,*], and Jianwan Ding[1]
[1]Huazhong University of Science and Technology, School of Mechanical Science and Engineering, Wuhan, 430074, China
*chenlp@hust.edu.cn

Two-Link Manipulator

In two-link manipulator simulation experiments, verification of the obtained mass matrices is shown in Table 1.

Table S1. Mass matrix property verification.

q(rad)	Model	$\|M - M^T\|_F$	Eigenvalues	Min(λ)
(-5,-1.57)	PHNODEs	0.0	[7.6522, 0.9779, 0.3589, 0.3026, 0.3000, 0.3000]	0.3000
	**SPEL**	0.0	[2.8618, 0.5076, 0.4487, 0.4487, 0.4487, 0.4487]	0.4487
	**SPEL-KAN**	0.0	[1.3356, 0.2045, 0.5986, 0.5986, 0.5986, 0.5986]	0.2045
(-4,1.57)	PHNODEs	0.0	[7.2470, 0.9758, 0.3540, 0.3023, 0.3000, 0.3000]	0.3000
	**SPEL**	0.0	[1.3329, 0.2049, 0.5986, 0.5986, 0.5986, 0.5986]	0.2049
	**SPEL-KAN**	0.0	[2.8168, 0.5153, 0.4487, 0.4487, 0.4487, 0.4487]	0.4487
(0,0)	PHNODEs	0.0	[8.1508, 0.9828, 0.3650, 0.3030, 0.3000, 0.3000]	0.3000
	**SPEL**	0.0	[1.6311, 0.1563, 0.5986, 0.5986, 0.5986, 0.5986]	0.1563
	**SPEL-KAN**	0.0	[3.7680, 0.3522, 0.4487, 0.4487, 0.4487, 0.4487]	0.3522
(0,1.57)	PHNODEs	0.0	[7.2314, 0.9748, 0.3541, 0.3023, 0.3000, 0.3000]	0.3000
	**SPEL**	0.0	[1.3917, 0.1954, 0.5986, 0.5986, 0.5986, 0.5986]	0.1954
	**SPEL-KAN**	0.0	[3.2620, 0.4390, 0.4487, 0.4487, 0.4487, 0.4487]	0.4390

[1] -KAN indicates that the internal MLP has been replaced with a KAN.
